# High-Accuracy HLA Type Inference from Whole-Genome Sequencing Data Using Population Reference Graphs

**DOI:** 10.1371/journal.pcbi.1005151

**Published:** 2016-10-28

**Authors:** Alexander T. Dilthey, Pierre-Antoine Gourraud, Alexander J. Mentzer, Nezih Cereb, Zamin Iqbal, Gil McVean

**Affiliations:** 1 Wellcome Trust Centre for Human Genetics, University of Oxford, Oxford, United Kingdom; 2 NHGRI-NIH, Bethesda, MD, United States of America; 3 Neurology Department, UCSF, San Francisco, United States of America; 4 Inserm unit 1064 ATIP-Avenir team 6, University of Nantes–Nantes University Hospitals, Nantes, France; 5 Histogenetics, Ossining, United States of America; 6 Li Ka Shing Centre for Health Information and Discovery, University of Oxford, Oxford, United Kingdom; Institute for Clinical Molecular Biology, GERMANY

## Abstract

Genetic variation at the Human Leucocyte Antigen (HLA) genes is associated with many autoimmune and infectious disease phenotypes, is an important element of the immunological distinction between self and non-self, and shapes immune epitope repertoires. Determining the allelic state of the HLA genes (HLA typing) as a by-product of standard whole-genome sequencing data would therefore be highly desirable and enable the immunogenetic characterization of samples in currently ongoing population sequencing projects. Extensive hyperpolymorphism and sequence similarity between the HLA genes, however, pose problems for accurate read mapping and make HLA type inference from whole-genome sequencing data a challenging problem. We describe how to address these challenges in a Population Reference Graph (PRG) framework. First, we construct a PRG for 46 (mostly HLA) genes and pseudogenes, their genomic context and their characterized sequence variants, integrating a database of over 10,000 known allele sequences. Second, we present a sequence-to-PRG paired-end read mapping algorithm that enables accurate read mapping for the HLA genes. Third, we infer the most likely pair of underlying alleles at G group resolution from the IMGT/HLA database at each locus, employing a simple likelihood framework. We show that HLA*PRG, our algorithm, outperforms existing methods by a wide margin. We evaluate HLA*PRG on six classical class I and class II HLA genes (HLA-A, -B, -C, -DQA1, -DQB1, -DRB1) and on a set of 14 samples (3 samples with 2 x 100bp, 11 samples with 2 x 250bp Illumina HiSeq data). Of 158 alleles tested, we correctly infer 157 alleles (99.4%). We also identify and re-type two erroneous alleles in the original validation data. We conclude that HLA*PRG for the first time achieves accuracies comparable to gold-standard reference methods from standard whole-genome sequencing data, though high computational demands (currently ~30–250 CPU hours per sample) remain a significant challenge to practical application.

## Author Summary

Determining an individual’s HLA type (the sequence of the exons of the HLA genes) is important in many areas of biomedical research. For example, HLA types shape immune epitope repertoires, which are relevant in cancer immunotherapy, and influence autoimmune and infectious disease risk. Whole-genome sequencing data, currently being generated for hundreds of thousands of individuals, contains the information necessary for HLA typing–but inferring accurate HLA types from these is a challenging problem. First, the HLA genes are the most polymorphic genes in the human genome; second, these genes and their variant alleles exhibit high degrees of sequence similarity (due to a shared evolutionary origin). This makes it difficult to establish which specific HLA gene a given observed sequencing read derives from. We show that this problem can be addressed using a Population Reference Graph (PRG): for each gene, the PRG contains not only the reference sequence but also variant alleles, thus enabling, using a novel sequence-to-graph mapping algorithm, the accurate mapping of reads to HLA genes. We also show that HLA*PRG, the algorithm implementing our approach, achieves–based on standard whole-genome sequencing data–accuracies comparable to those of specialized gold-standard methods. HLA*PRG is open source and freely available.

## Introduction

Genetic variation at HLA loci, both classical and non-classical, is associated with many medical phenotypes including risk of autoimmune [1–3] and infectious [4] disease, adverse drug reactions [5, 6], success of tissue and organ transplants [7], and, via epitope presentation preferences, the success of cancer immunotherapy [8]. The current gold standard for high resolution typing of HLA alleles, sequence-based typing (SBT), uses Sanger sequencing or targeted amplification of the HLA genes followed by next-generation sequencing. With the growth of high throughput genomic technologies, methods for inferring HLA genotype have been developed that use SNP genotyping [9–12] or next-generation sequencing [13–19]. These approaches, to date, are either limited to a subset of HLA loci, require targeted capture / amplification, or do not achieve the same degree of accuracy as SBT.

The main challenge in characterising the HLA genes from next-generation sequencing data is the correct alignment of sequencing reads. Multiple factors influence accuracy, including the sheer sequence and structural diversity of the region, the presence of multiple paralogous genes (including pseudogenes) and rare, but important, gene conversion events that generate mosaic allelic structures. The high degree of sequence similarity between alleles in certain groups of loci and its non-random spatial structure are illustrated in S1 Fig and S2 Fig.

To address these challenges, we have previously introduced structures to represent known genomic variation called population reference graphs (PRGs) and demonstrated their value in characterising variation across the major histocompatibility complex (MHC) and particularly within the HLA class II gene region [20]. Briefly, a PRG is a directed graph in which alternative alleles, insertions and deletions are represented as alternative paths through the graph, and in which orthologous and identical regions are collapsed locally to model potential recombination. Previously, we demonstrated that a prototype of this approach could identify the nucleotide-level variants at classical HLA alleles with high accuracy. However, we did not address the problem of inferring the alleles present at the gene level [20].

We set out to extend the PRG approach to provide accurate HLA typing at G group resolution (see below) using high coverage whole-genome sequencing data, such as is being generated by large-scale genomics projects. This study presents novel developments in 3 main areas:

First, we build a gene-only PRG that combines genomic haplotypes (spanning the complete MHC), gene haplotypes and exon sequences for 46 (mostly HLA) genes (S1 Table). In our previous work we utilized a whole-MHC PRG and didn’t attempt to integrate exon sequences. A gene-only PRG is smaller and therefore computationally advantageous, and integration of the exon sequences gives a more comprehensive model of genetic variation at the HLA loci.

**Fig 1:**
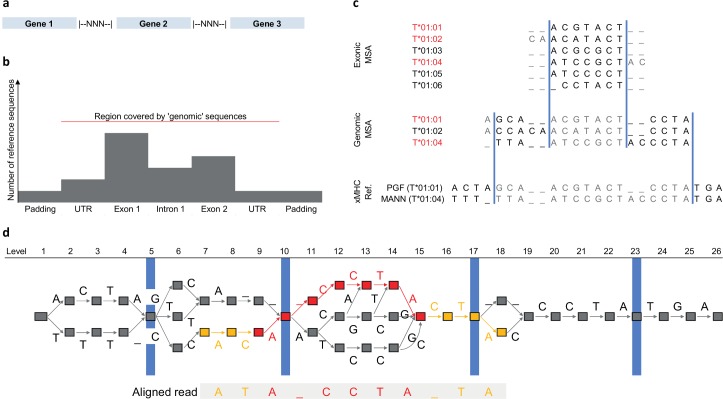
Schematic representation of HLA type inference using HLA*PRG. **a** Broad-scale structure of the HLA PRG. The included genes are separated by spacer blocks consisting of N characters. **b** Fine-scale structure of the PRG input sequences. Exons, introns and UTRs are embedded in regional haplotypes (padding sequence). Exon sequences typically outnumber intron sequences. The red line indicates the region covered by IMGT genomic sequences. X-axis not to scale. **c** For each gene represented in the PRG, multiple sequence alignments representing up to 3 sources of sequence data are merged for PRG construction: exonic sequences, genomic (UTR, exons, introns) sequences, regional haplotypes (“xMHC Ref.”). Using alleles present in both the current and the next-higher-level MSA (identifiers printed in red), the merging algorithm determines consensus boundaries (blue bars) to connect the MSAs of different input sequence types. For each segment so-defined, we use the MSA corresponding to the highest-resolution input sequence type (sequence characters therefore ignored are printed in grey). **d** The PRG corresponding to the input sequences shown in c, and a seed-and-extend alignment of a sequencing read to the PRG. PRG nodes are represented by boxes and edges by labelled arrows. The four blue markers correspond to the consensus MSA boundaries shown in c. The aligned sequence of the read is displayed below the PRG, and the alignment path (the sequence of edges and nodes traversed in the PRG) is highlighted. The red component of the alignment path corresponds to the exact-match “seed” component of the alignment (spanning a graph-encoded gap), whereas the orange components correspond to the “extend” component of the alignment (where mismatches are allowed).

Second, we present an algorithm to map short (e.g. 100 or 250bp) paired-end next-generation sequencing reads directly to the PRG. We had previously [20] described an approach for long non-paired reads. The short-read algorithm we present here follows a classical seed-and-extend paradigm; that is, it identifies areas of exact identity between the graph and the read to be mapped, and tries to extend these using dynamic programming, allowing for mismatches, insertions and deletions. Each alignment follows a possible walk through the PRG. Importantly, because the PRG encodes information on sequence variation and because the mapping algorithm utilizes this information, it enables accurate alignment in the presence of homologous variants and a more precise quantification of mapping ambiguity. The short read mapping algorithm is relatively slow and benefits from our decision to limit ourselves to a gene-only PRG. See Fig 1 for a schematic depiction of graph topology and read mapping.

Third, conditional on reads mapped to the PRG and a database of possible underlying haplotypes (i.e., the HLA allele sequences), we infer the most likely pair of underlying haplotypes and a quality score using a simple likelihood framework.

We implement our approach in an open source package called HLA*PRG and show in two validation experiments that the level of achieved accuracy is comparable to SBT. Allelic variants at HLA genes can be typed at different degrees of resolution; low resolution (“1-field”, formerly “2-digit”) types specify serological activity; intermediate resolution (“2-field”; formerly “4-digit”) HLA types specify the complete primary sequence of the HLA proteins and high-resolution (“3-field”; formerly “6-digit”) types determine the full exonic sequence including synonymous variants. Higher levels of resolution include non-coding variation. SBT is typically carried out at “G group” resolution, in which only the sequences of the exons encoding the peptide binding groove are considered: exons 2 and 3 for HLA class I genes and exon 2 for HLA class II genes. In most applications of typing, a set of 6–8 loci are typed (Class I: *HLA-A*, *-B*, -C, Class II: *HLA-DQA1*, -*DQB1*, -*DRB1*, -*DPA1* and -*DPB1*), though there exist over 30 HLA genes and pseudogenes.

Like SBT, HLA*PRG reports HLA types at G group resolution. Lower-resolution types are only used when benchmarking against other HLA type inference algorithms that fall back to these in cases of ambiguity.

## Results

To assess the accuracy of HLA*PRG, we use two data sets with available high coverage sequencing data and independent SBT-based HLA type information for 6 classical class I and class II loci (Table 1).

**Table 1:** HLA type inference accuracy for HLA*PRG and two state-of-the-art algorithms.

Cohort	Locus	N^a^	HLA*PRG			PHLAT			HLAreporter		
			Inferred	Accuracy	Call Rate	Inferred	Accuracy	Call Rate	Inferred	Accuracy	Call Rate
**Platinum Trio**	A	6	6	1.00	1.00	6	1.00	1.00	2	0.50	0.33
	B	6	6	1.00	1.00	6	1.00	1.00	1	1.00	0.17
	C	6	6	1.00	1.00	6	1.00	1.00	1	0.00	0.17
	DQA1	6	6	1.00	1.00	6	1.00	1.00	2	1.00	0.33
	DQB1	6	6	1.00	1.00	6	1.00	1.00	5	1.00	0.83
	DRB1	6	6	1.00	1.00	6	1.00	1.00	5	1.00	0.83
**1000 Genomes Highest Resolution**	A	22	22	1.00	1.00	20	0.45	0.91	0	NA	0.00
	B	22	22	1.00	1.00	20	0.35	0.91	6	0.50	0.27
	C	22	22	1.00	1.00	20	0.50	0.91	2	0.50	0.09
	DQA1	12	12	1.00	1.00	10	0.70	0.83	9	1.00	0.75
	DQB1	22	22	1.00	1.00	20	0.80	0.91	15	1.00	0.68
	DRB1	22	22	0.95	1.00	20	0.55	0.91	10	1.00	0.45
**1000 Genomes 2-field resolution**^b^	A	22	22	1.00	1.00	20	0.70	0.91	0	NA	0.00
	B	22	22	1.00	1.00	20	0.60	0.91	6	0.50	0.27
	C	22	22	1.00	1.00	20	0.80	0.91	2	0.50	0.09
	DQA1	12	12	1.00	1.00	10	0.70	0.83	9	1.00	0.75
	DQB1	22	22	1.00	1.00	20	0.95	0.91	15	1.00	0.68
	DRB1	22	22	0.95	1.00	20	0.75	0.91	10	1.00	0.45

^a^ Number of validation alleles.

^b^ “1000 Genomes Highest Resolution” and “1000 Genomes 2-field resolution” represent the same set of samples, with G group alleles (available for 9/12 samples) reduced to 2-field resolution for the latter experiment, enabling a fair comparison with algorithms that fall back to 2-field typing in cases of ambiguity.

First, we analyse NA12878, NA12891 and NA12892 from the Illumina Platinum Genomes Project, sequenced to 50 - 55x with a PCR-free 2 x 100bp protocol. We correctly infer all 36 HLA alleles.

Second, we analyse 11 samples from the 1000 Genomes Project, sequenced to 28 – 68x with a PCR-free 2 x 250bp protocol. In terms of diversity, the 11 samples represent 7 ethnicities (S2 Table); a wide range of HLA types, e.g. 9 different 1-field groups for HLA-DRB1 (S2 Table); and heterozygous as well as homozygous loci (S3 Table). Initial analysis identifies three discrepancies (S1 Text), though on re-typing these individuals two of three are the result of initial errors in the validation data. The remaining inconsistency, (HLA-DRB1*16:*02*:*01* incorrectly typed as HLA-DRB1*16:*23*) is likely caused by *HLA-DRB5* sequences incorrectly aligned to *HLA-DRB1* (IMGT/HLA, the HLA sequence database, currently doesn’t contain genomic sequences for HLA-DRB5 and the representation of this gene in the PRG therefore remains incomplete).

We compare the performance of HLA*PRG with PHLAT [14] and HLAreporter [13], two state-of-the-art algorithms that support HLA class I and class II (Table 1). For the Platinum samples, we find that PHLAT also correctly infers all 36 alleles, whereas HLAreporter only reports 16 alleles (of which 14 are correct). For the 1000 Genomes Samples, we find that HLA*PRG outperforms both programs by a wide margin. Mean accuracy at 2-field resolution across all loci is 75% for PHLAT and 80% for HLAreporter, and HLAreporter achieves a call rate of only 38%. To confirm that the observed differences in performance are not merely driven by different approaches to encoding ambiguous alleles, we repeat the 1000 Genomes validation experiment at 1-field resolution, the lowest and most ambiguous level of HLA typing; at 100% accuracy, HLA*PRG remains ahead of PHLAT and HLAreporter, which achieve accuracies of 89% and 90%, respectively (S4 Table).

To evaluate sensitivity of HLA*PRG to whole-genome sequencing depth, we subsampled the NA12878 data from the Platinum and 1000 Genomes projects to average coverages of 40x, 30x and 20x in triplicates. We find that performance is stable (all alleles correctly predicted) down to 20x for the Platinum data and down to 30x for the 1000 Genomes data (S5 Table).

To assess to what extent HLA*PRG depends on the availability of whole-genome data, we carried out two additional experiments. First, we apply HLA*PRG to whole-exome sequencing data of a cohort of HapMap samples. Results are varied and accuracies consistently lower across all loci (ranging from 79% for *HLA-C* to 98% for *HLA-DQB1*, S6 Table). Second, we apply HLA*PRG to a cohort of 14 Ugandan samples that underwent targeted amplification and MiSeq-based high coverage sequencing of the HLA exons (this cohort also contains a novel *HLA-B* and a novel *HLA-DQB1* allele, see below). Average accuracy is 95% at G group resolution (PHLAT: 74%; HLAreporter: 73%) and 96% at 2-field resolution (PHLAT: 97%; HLAreporter: 80%) (S7 Table). Of the 6 erroneous alleles at 2-field resolution, 2 are novel alleles; an additional 2 errors are associated with mis-inferred DRB1*14:141 alleles, which are exon 2-identical to DRB3 sequences (which we expect, due to linkage with DRB1*11/13 alleles, also to be present in the samples).

For each inferred allele, HLA*PRG reports three quality statistics: a parametric likelihood-based quality score (S1 Text) and the proportion of k-Mers associated with the allele present in the sample sequencing data, both ranging from 0 to 1; and the number of columns in the read-to-graph alignment that contain alleles with an allele frequency ≥0.2 that are not accounted for by the diploid HLA call for the sample (“unaccounted alleles”). For the first two metrics, samples with lower values are enriched for errors; there is, however, no clear cut-off between correct and incorrect alleles (S3 Fig). The two novel alleles cluster with the bulk of correct calls. For the “unaccounted alleles” statistic, we observe that the number of columns with high-frequency unaccounted alleles varies systematically between loci (S4 Fig), and that there is no systematic difference between correct and incorrect calls. Although the two novel alleles score comparatively highly on this metric, it doesn’t enable the clear distinction between novel and non-novel alleles (S4 Fig). Nevertheless, the combination of these quality metrics can help identify alleles with higher uncertainty.

To assess whether HLA*PRG could be applied to additional HLA loci beyond the set of the 6 classical genes validated here, we use it to genotype a set of 12 additional HLA genes and pseudogenes in the two trios that are part of our whole-genome cohorts (S8 Table). Across the 2 x 72 alleles inferred, we find one trio inconsistency in the CEU trio (pseudogene *HLA-K*, driven by an allele called with low confidence)); and two inconsistencies in the YRI trio (the *HLA-DRB1* inconsistency described above and an additional inconsistency at *HLA-K*).

## Discussion

We have shown that HLA*PRG enables HLA typing from standard whole-genome next-generation sequencing data at accuracies comparable to those of the current gold-standard SBT technology (two errors in the original reference data compared to one from HLA*PRG at 2-field / G group resolution)–provided that high-quality whole-genome sequencing data are used as input (PCR-free protocol, read length of at least 100bp, coverage of at least 30x).

Importantly, our results apply to both the established 2 x 100bp and the more recent 2 x 250bp Illumina HiSeq protocols; they are therefore directly applicable to many of the large-scale sequencing projects currently ongoing. HLA*PRG will enable researchers to augment these cohorts with reliable HLA type information and can contribute to characterizing HLA signals in important medical phenotypes.

The current implementation of HLA*PRG is limited in three respects.

First, although the algorithm was designed explicitly for application to high-quality whole-genome sequencing data, it would be advantageous if comparable performance was achieved from other data sources. Our evaluation shows that this is not the case. The exome sequencing cohort exhibits the lowest accuracy of all cohorts examined; it is also the cohort with the lowest effective fragment length (2 x read length + insert size, S5 Fig). High effective fragment lengths help overcome local sequence homologies like those observed in the HLA region, and it is likely that this factor plays a role in explaining the poor performance of the exome cohort. Alignment issues also likely contribute to the slight reduction in accuracy observed when applying HLA*PRG to the MiSeq sequencing data (exon targeting by PCR) validation cohort. Although the effective fragment length of this cohort is higher, the vast majority of reads start and end within a few bases of the exon boundaries. In comparison to whole-genome data, where the majority of exon-spanning reads run into introns and the variation contained therein, this exacerbates the effects of exon sequence homologies between different genes (consistent with the observation that the problem of HLA*PRG mis-inferring a small number of alleles due to DRB1-DRB3 distinction issues arises only in the MiSeq, but not in the whole-genome, cohort). Of note, base quality doesn’t seem to strongly influence accuracy; when measured by the number of read bases agreeing with the graph location they are aligned to, average base quality is lowest for the 1000 Genomes cohort (87%) and highest for the exome cohort (99%). In summary, high (≥30x) uniform coverage across the whole length of the HLA genes and high fragment lengths seem to deliver best results; caution should be exercised when these conditions are not met (e.g. for targeted amplification, whole-genome amplification, targeted capture).

**Fig 2:**
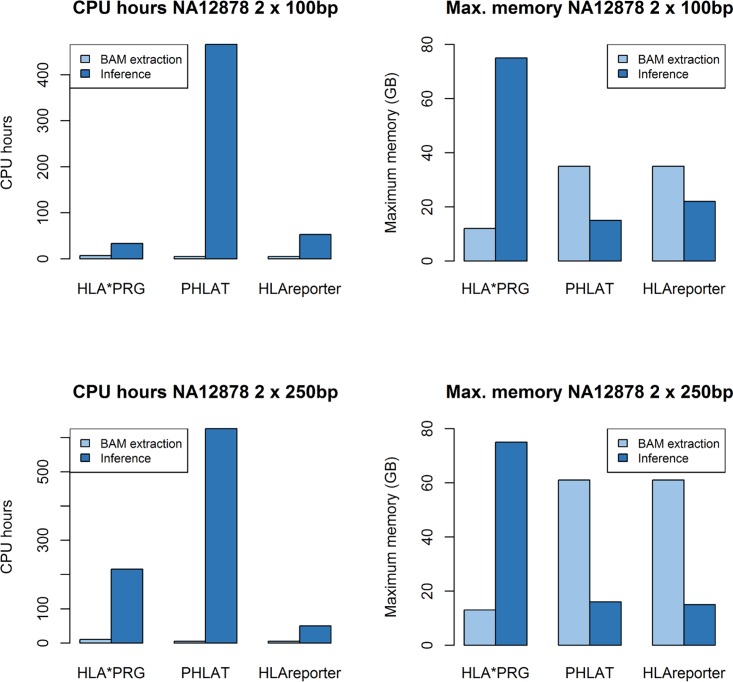
Runtime and memory requirements comparison of HLA*PRG, PHLAT and HLAReporter on NA12878. Upper part: NA12878 2 x 100bp reads from the Platinum cohort; lower part: NA12878 2 x 250bp reads from the 1000 Genomes cohort. We provide a detailed analysis in S1 Text.

Second, HLA*PRG is optimised for accuracy rather than computational efficiency. Analysing the NA12878 data takes between 33 and 215 CPU hours (Platinum / 1000G data; AMD Opteron 6174 2.2GHz; 11–17 hours clock time). Analysing the same data with PHLAT and HLAreporter takes 466/626 and 53/50 CPU hours, respectively (Fig 2). Depending on the availability of high-performance compute infrastructure and the number of samples to analyse, computational demands might represent a significant barrier to adoption. We provide a detailed runtime (including CPU time) and memory analysis in S1 Text. Achieving improvements in computational efficiency is ongoing work, but it is worth noting that HLA*PRG can be run immediately after the raw sequence data has been mapped, in parallel with standard variant-calling. Future versions might make use of linear sequence alignments to seed graph alignment (similar to ALT-aware alignment in BWA-MEM[21]) and also incorporate population haplotype frequencies [22, 23].

Third, HLA*PRG doesn’t enable the discovery of new alleles and it is limited to G group resolution. It would be advantageous if it was possible to identify samples that harbour novel alleles from the quality metrics of the inferred alleles. This, however, was not the case for the two novel alleles in the Ugandan cohort and the 3 parametric and non-parametric statistics we analysed. It seems (S4 Fig) that there is a residual number of reads aligned to the wrong gene, and distinguishing between the signal generated by these and that of true novel alleles is non-trivial. Further research will be necessary to address this.

The good performance of HLA*PRG is consistent with the hypothesis that reference graph approaches are well-suited to enable accurate genome inference in highly complex, highly diverse regions of the human genome. At the example of the MHC as a whole, we had already shown that this was the case using small-scale (SNPs, k-Mers) and large-scale (long reads) metrics of genome inference accuracy [20]. By focusing on G group resolution HLA typing, this study complements the existing evidence with another important metric: exon-scale haplotype accuracy. Faster algorithms for sequence-to-graph alignment than the one used here are currently under development (https://github.com/vgteam/vg) and these will likely simplify the development of future variation graph-based approaches.

There are other regions in the human genome that could benefit from a tailored PRG-based inference approach. One example is the LRC/KIR region on chromosome 19, which is similar to the MHC in some aspects (hyperpolymorphism, availability of haplotype and allele databases[24]) and different in others (higher degrees of structural variation and paralogy [25–27]). One important implication of the results presented here is that a variation-aware read mapping algorithm that processes reads independently (in the sense that no attempt at local haplotype reconstruction is made *during* the read mapping process) is sufficiently accurate for HLA genotyping. Whether this also applies to the LRC/KIR region is an open question.

## Materials and Methods

In this Section we present a high-level summary of PRG construction, read mapping, HLA type inference and validation. A full technical description of the algorithms is given in S1 Text.

### A Population Reference Graph for the HLA genes

We construct a gene-only PRG for 46 genes from 720 genomic sequences (from IMGT [28] / GRCh37), 10050 exonic sequences (from IMGT) and 8 MHC haplotypes (from GRCh37). For each gene independently, we combine (see next Section) exonic sequences (where available), genomic sequences and 0.5kb of surrounding non-genic “padding” sequence from the 8 MHC haplotypes (where appropriate; to enable the alignment of reads that span a gene boundary). We then construct a joint PRG [20], in which we separate genes with 2000 N characters (see Fig 1A for a schematic depiction of the topology of the PRG created).

### MSA merging algorithm

We employ a multiple sequence alignment (MSA) merging algorithm (Fig 1C) to combine genomic, exonic and genomic “padding” sequences for PRG construction. Typically there are more exonic sequences than genomic sequences, and more genomic sequences than genomic “padding” sequences (Fig 1B).

We describe the base case of merging the MSA for one exon into the MSA of surrounding genomic sequences. The other cases follow immediately and are described in S1 Text.

The aim of the MSA merging algorithm is to find the “switch points” between the exon MSA and the genomic MSA; i.e. the coordinates at which the PRG switches from one MSA to the other and back (blue lines in Fig 1C). To compute the switch points we rely on shared alleles (i.e. alleles that are present in both MSAs)–for each shared allele, we should be able to identify the exon sequence as a substring of the genomic sequence alignment, defining the coordinates of the exon MSA in genomic MSA terms. If there is more than one shared allele, we compute consensus switch points.

Switch coordinate computation for the exon MSA:

1. Initialize *P*_*L*_ = { } and *P*_*R*_ = { }. These two sets hold the coordinates of the allele-specific left and right switch points in the exon MSA.
2. Allele-specific switch points: For each shared allele, add the exon MSA coordinates of the beginning and the end of the un-aligned exon allele sequence to *P*_*L*_ and *P*_*R*_, respectively (that is, the coordinates of the un-aligned sequence in the alignment—for example, if an exon MSA sequence is–-ACGT…, due to the two gaps at the beginning of the alignment, we add the value 3 to *P*_*L*_).
3. Define the consensus switch coordinates as *G*_*L*_ = max(*P*_*L*_) and *G*_*R*_ = min(*P*_*R*_).

The steps as described above define the area (from coordinates *G*_*L*_ to *G*_*R*_) of the exon MSA to be utilized for PRG construction. For each individual *genomic* shared allele sequence, this leaves sequence to the left and to the right of the extracted exon sequence. We combine all such ‘left’ (genomic) sequences from all shared alleles and compute [29] a new MSA; we also combine all such ‘right’ (genomic) sequences from all shared alleles and compute a new MSA. Finally, the two MSAs so-created are used as the segments to the left and to the right of the extracted exon MSA block for PRG construction (Fig 1B).

A full description of the PRG construction algorithm is given in S1 Text and the output data are part of the HLA*PRG data package (available on the HLA*PRG website).

### Read alignment

#### Read-to-graph alignment

Basic sequence-to-PRG alignment algorithms for long, non-paired reads were described in [20]. Here we present a modified algorithm optimized for short reads (Fig 1D):

1. For each read of a read pair, find regions of exact matches (highlighted in red in Fig 1D) between the read sequence and walks in the graph. This step utilizes a k-Mer index of the graph, and chains together k-Mers that are connected in both graph and read sequence. After the exact match step, for each identified match, locally extend the exact match region using the alignment algorithms presented in [20] (highlighted in orange in Fig 1D).
2. For each read pair, the total number of possible alignments is given by multiplying the number of possible alignments for read 1 by the number of possible alignments for read 2 (identified during the previous step).
3. We score each such paired-end alignment in a likelihood framework, taking into account the individual alignment scores, read pair orientation and insert size characteristics. By normalizing we obtain a probability distribution over possible paired-end alignments from normalization.
4. In a final step, we obtain the maximum-likelihood alignment (which we treat as fixed for all downstream analyses); a quality score for this alignment; a per-position alignment quality score that measures how confidently we align a particular base in the read to a particular level in the graph.

We give a full technical description of the alignment algorithm in S1 Text.

We highlight two differences to the algorithm utilized in [20]. First, the algorithm presented here uses paired-end information. Second, the extension stage as defined here starts from *one* region of exact match and terminates if it reaches the end of the read or other termination conditions; the algorithm in [20], in contrast, is based on the notion of using extension steps to connect *multiple* regions of exact matches.

#### Read filtering

As read alignment (in our current implementation) is computationally demanding, we employ a pre-filtering step to identify reads that likely emanate from regions covered by the PRG. Only reads that pass this filter are aligned to the PRG.

In the standard version of the filter, we keep read pairs that

- have >30% k-Mers present in the PRG (positive selection).
- have ≥1 reads with ≥1 k-Mers unique to the PRG, or that have ≥1 reads with <45% k-Mers present in genomic regions outside the PRG (negative selection).

We use modified criteria for reads with a higher proportion of low-quality positions, such as the 2 x 250bp reads from the 1000 Genomes Project samples. Full details are given in S1 Text.

### HLA type inference

Let *R* be the set of paired-end read alignments that overlap with the peptide-binding site of a given HLA locus. We calculate the likelihood of the observed data *R* conditional on pairs of possible underlying HLA types at G group resolution. We note that each HLA type (i.e., each possible underlying allele) has a defined genotype (potentially including “gap” characters) at each position of the peptide binding site as represented in the PRG.

For an arbitrary pair (*a*_1_,*a*_2_) of underlying alleles, we define the likelihood functions

$$\text{L}(R|\left(a_{1},a_{2}\right))≔\prod_{r\in R}\text{L}(r|\left(a_{1},a_{2}\right))\;and$$

$$\text{L}(r|\left(a_{1},a_{2}\right))≔\frac{1}{2}\times \text{L}(r|a_{1})+\frac{1}{2}\times \text{L}(r|a_{2}).$$

L(*r*|*a*) is the likelihood of observed aligned read pair *r*, conditional on an assumed underlying HLA allele *a*.

By definition *r* overlaps with the peptide-binding site. At each overlapping position, we have a pair (*g*_*r*_,*g*_*a*_), where *g*_*r*_ is the nucleotide (or gap) of the aligned read *r* (and its associated base quality, if applicable) at this position, and *g*_*a*_ is the genotype of underlying HLA allele *a* at this position. We define the set *O*_*r*_ as the set of pairs for all overlapping positions of *r*.

Finally, we define

$$\text{L}(r|a)≔\prod_{(g_{r},g_{a})\in O_{r}}\text{score}(g_{r},g_{a})$$,

with score being a base-quality-aware alignment scoring function for matches, mismatches, deletions and insertions.

We compute the likelihood function over all pairs of possible underlying alleles and normalize to obtain a probability distribution over possible underlying allele pairs. We call two “best guess” alleles and their associated qualities as described in [10]. Briefly,

- to obtain the first “best guess” allele we select the allele with the highest probability of occurring ≥1 times. We use the marginal probability as quality score.
- to obtain the second “best guess” allele we consider all pairs that contain the first best-guess allele and select the pair with the highest absolute probability. We use the absolute pair probability as quality score for the second allele.

We give a full technical description of the likelihood-based inference procedure in S1 Text.

### Validation data

#### Sequencing data

We validate HLA*PRG on 2 sets of validation samples with publicly available high coverage whole-genome sequencing data.

First, NA12878, NA12891 and NA12892, which were whole-genome sequenced for the Illumina Platinum Genomes Project (50 - 55x with a PCR-free 2 x 100bp protocol).

Second, 11 samples from the 1000 Genomes Project [30], which were whole-genome sequenced to high coverage (28 – 68x with a PCR-free 2 x 250bp protocol).

To assess the extent to which accurate HLA typing with HLA*PRG depends on the availability of whole-genome data, we also evaluate it on a cohort of 29 exome-sequenced HapMap [31] samples (also sequenced for the 1000 Genomes Project) and on a cohort of 14 MiSeq-sequenced Ugandan individuals. Sequencing data for the Ugandan individuals is available upon request (see below).

We list data download URLs and accession IDs for all utilized sequencing data in S1 Text.

### HLA types

Except for the Ugandan MiSeq cohort, HLA types for all validation samples are either publicly available [32, 33] or available from a previous study [10].

14 samples from Ugandan individuals were available through the Entebbe Mother and Baby Study courtesy of Alison Elliott[34]. DNA was extracted from EDTA-stored cell pellets using the QIAamp DNA Blood Mini Kit (QIAGEN, Germany) before undergoing SBT using two methods. The first method, a Sanger-based approach for validation, was undertaken at Addenbrooke’s Tissue Typing Laboratory, (Cambridge, UK) using the Fisher Scientific proprietary uTYPE (version 7) software (Fisher Scientific, MA, USA). Exon-targeted MiSeq sequencing was undertaken at Histogenetics (NY, USA) using proprietary protocols. After validating the MiSeq-based calls with the Sanger data, the MiSeq data were used for validation. All data will be made available to interested researchers upon request through the African Partnership for Chronic Disease Research Data Access Committee.

S1 Text contains a table of utilized samples and their HLA types.

### Software implementation

HLA*PRG is implemented in C++/Perl and available under GPLv3 as part of the MHC*PRG repository https://github.com/AlexanderDilthey/MHC-PRG. A readme file (https://github.com/AlexanderDilthey/MHC-PRG/blob/master/HLA-PRG.md) describes how to install and run the software. A compiler with support for C++11 and openMP is required (e.g., GCC 4.7.2). We provide a separate HLA*PRG data package (download size ~41G), independent from the larger MHC*PRG data package.

## Supporting Information

S1 Fig **Sequence homology between HLA-A, -B and–C.** Graphs visualizing sequence homology between *HLA-A*, *-B* and *-C* across exons 2 (left) and 3 (right), based on an IMGT/HLA-provided multiple sequence alignment (MSA) of 3284 -A, 4077 -B, 2799 -C alleles. The x axis of the plot represents the column index of the MSA (304 columns for exon 2, 349 columns for exon 3). The (invisible) nodes of the graph represent the set of unique 31-mers (across the 3 genes) starting at the corresponding column of the MSA. Two nodes (representing two consecutive 31-mers in the MSA) are connected by (visible) edges if the corresponding 32-mer, starting at the column index of the first 31-mer, is present in the MSA. Edge flow (line thickness) is proportional to the frequency of the corresponding 32-mer at the underlying column (bounded below). Edge colour indicates the proportions of flow attributable to the 3 genes (for each edge, the absolute count of the corresponding 32-mer at the underlying column can be split into a triplet representing the *HLA-A*, *HLA-B*, *HLA-C* rows of the alignment; the (R, G, B) colour of the edge is obtained by normalizing this triplet). For the purpose of this plot, we treat gap characters as nucleotides. The plots below the graphs show, separately for *HLA-A*, *-B*, and–*C* and separately for each column of the underlying MSA, the (weighted) proportion of 31-mers unique to the locus. For the purpose of these plots, a k-Mer is defined as unique to a locus if it doesn’t occur in the same MSA column of a sequence belonging to another locus. k-Mer weights for each plotted column are proportional to within-locus k-Mer column frequencies.
(PNG)

S2 Fig Maximum HLA gene sequence homology at the peptide binding site. Maximum k-Mer similarity at the peptide binding site (PBS; exons 2/3 for HLA class I, exon 2 for HLA class II) between alleles of different HLA loci, based on k-Mers (k = 25). G group types are defined by PBS sequence. Each cell, in row X and column Y, contains the maximum, over all alleles of locus X, proportion of k-Mers present in any allele of locus Y. This quantity influences the probability of mismapping a PBS read to another locus as exact matching is the first step of the many mapping algorithms, including the one used here.
(PDF)

S3 Fig Allele quality score and allele k-Mer coverage for all validated alleles samples. Allele quality score and allele k-Mer coverage at k = 31 for all validated alleles (jittered). The colour of the data points indicates whether an allele was inferred correctly or incorrectly, or whether it was a novel allele (which are counted as ‘incorrect’ for all validation purposes).
(TIFF)

S4 Fig Number of columns with high-frequency unaccounted-for alleles. This plot shows, for each inferred allele and stratified by locus, the number of columns in the read-to-graph alignment that contain high-frequency (allele frequency ≥0.2) alleles not accounted for by the inferred (diploid) HLA type. The colour of the data points indicates whether an allele was inferred correctly or incorrectly, or whether it was a novel allele (which are counted as ‘incorrect’ for all validation purposes).
(TIFF)

S5 Fig Insert sizes, effective fragment lengths, validated alleles, and errors across cohorts. The figure shows how insert size and effective fragment length (2 x read length + insert size) differ between cohorts and correctly / incorrectly inferred alleles.
(PDF)

S1 Table HLA PRG input sequences. Loci represented in the HLA PRG. “Genomic alleles”: Genomic alleles represented in the gene-specific segment of the PRG, i.e. alleles spanning the complete length of the gene. “Exonic alleles”: Exonic alleles represented in the gene-specific fragment of the PRG.
(XLSX)

S2 Table Ethnicities and 1-field allele groups represented in the whole-genome validation data. Sample ethnicity and sample HLA type (1-field resolution) in the Platinum and 1000 Genomes whole-genome validation cohorts.
(XLSX)

S3 Table Validation cohort homozygosity statistics. Number of homo- and heterozygous validation samples, per locus and per validation cohort.
(XLSX)

S4 Table 1-field validation accuracy for the 1000 Genomes cohort. HLA type inference accuracy, measured at 1-field resolution, for HLA*PRG and two state-of-the-art algorithms, PHLAT and HLAreporter, on the 1000 Genomes whole-genome validation cohort (description see main text).
(XLSX)

S5 Table Coverage sensitivity analysis. Sensitivity to reduced coverage. Results for NA12878 (Platinum and 1000 Genomes data, see main text), down-sampled to 40x, 30x, 20x (triplicates).
(XLSX)

S6 Table Performance on exome sequencing data. HLA type inference accuracy, per locus, for HLA*PRG and two state-of-the-art algorithms, PHLAT and HLAreporter, on a set of exome-sequenced HapMap samples (2 x 100bp, average per-locus coverage at the peptide-binding site 54x (over all validated HLA loci and samples, minimum 4.4x, maximum 164x). “Highest Resolution” and “2-field resolution” represent the same set of samples, with G group validation alleles (where available) reduced to 2-field resolution for the latter experiment. Note that the number of inferred alleles varies between algorithms.
(XLSX)

S7 Table Performance on MiSeq sequencing data. HLA type inference accuracy, per locus, for HLA*PRG and two state-of-the-art algorithms, PHLAT and HLAreporter, on a set of MiSeq-sequenced samples from Uganda. The cohort contains a novel HLA-B15 allele and a novel HLA-DQB1*02 allele. “Highest Resolution” and “2-field resolution” represent the same set of samples, with G group validation alleles (where available) reduced to 2-field resolution for the latter experiment. Note that the number of inferred alleles varies between algorithms.
(XLSX)

S8 Table Trio consistency for additional HLA loci. HLA types for the CEU Platinum trio and for the YRI 1000 Genomes trio, including additional loci and typing quality scores. Genes from S3 Table (list of all genes in the PRG) not appearing here are not antigen-presenting or cannot be typed for technical reasons (no IMGT exon data available or incomplete resolution of the exon-to-genomic, genomic-to-haplotype alignment steps during PRG construction). HLA-DRB3 and HLA-DRB4 copy numbers are variable and linked to DRB1 genotype (neither aspect is modelled by HLA*PRG). Assumedly absent alleles (as determined by linkage with the inferred DRB1 alleles) are shaded in grey, and we note that these carry low quality scores. In the CEU trio, we detect one trio inconsistency at the HLA-K pseudogene (shaded in bright red), and note that the allele driving the inconsistency carries a low quality score; in the YRI trio, we detect two inconsistencies (the DRB1 inconsistency described in the main text and another inconsistency at HLA-K).
(XLSX)

S1 Text Full description of the HLA*PRG algorithms and validation data. (DOCX)
