# Supplementary figures and images for: High-Accuracy HLA Type Inference from Whole-Genome Sequencing Data Using Population Reference Graphs
Source: PLoS Comput Biol. 2016 Oct 28;12(10):e1005151. doi: 10.1371/journal.pcbi.1005151 (PMC5085092; doi:10.1371/journal.pcbi.1005151)

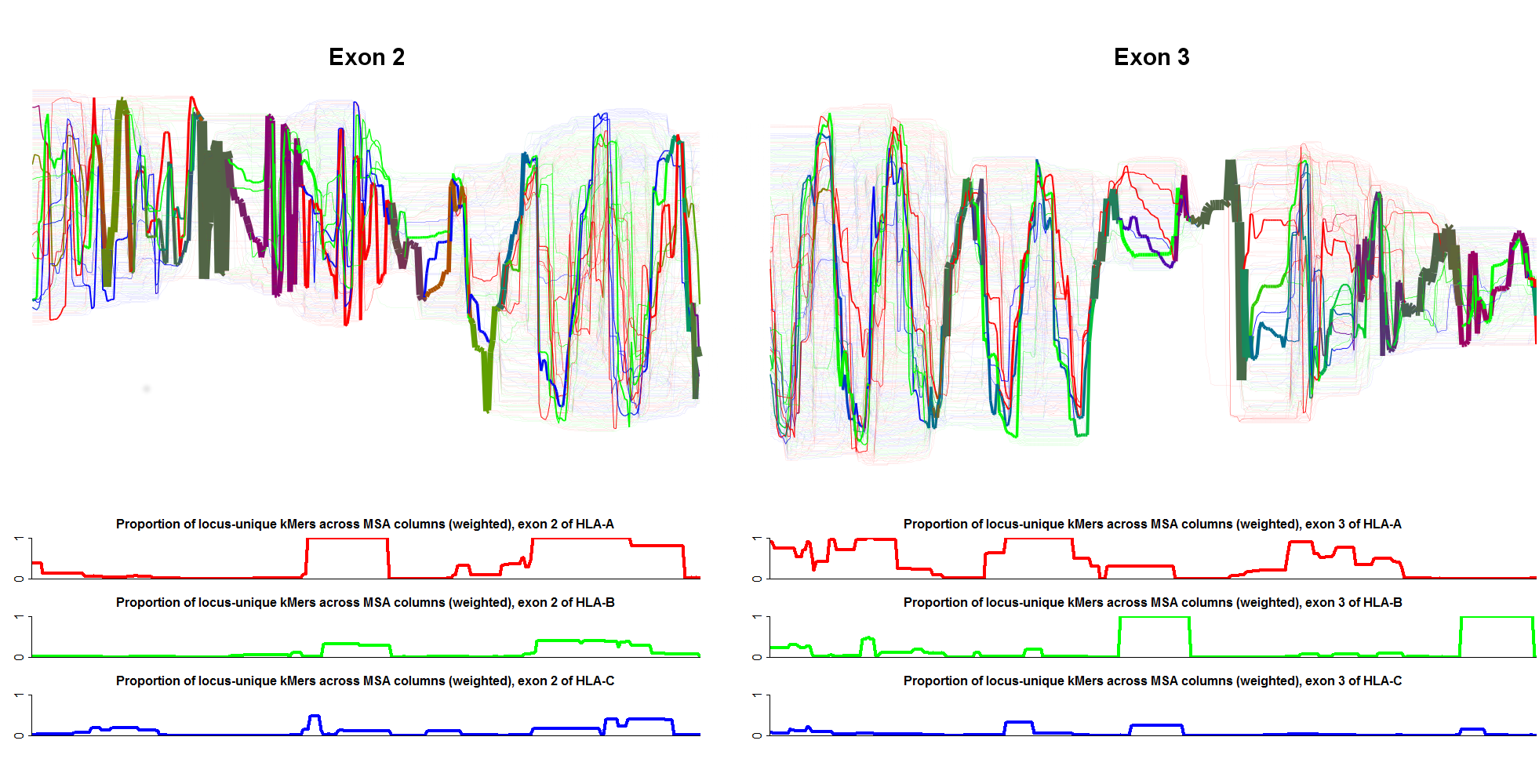

Supplement: S1 Fig — Sequence homology between HLA-A, -B and–C. Graphs visualizing sequence homology between HLA-A, -B and -C across exons 2 (left) and 3 (right), based on an IMGT/HLA-provided multiple sequence alignment (MSA) of 3284 -A, 4077 -B, 2799 -C alleles. The x axis of the plot represents the column index of the MSA (304 columns for exon 2, 349 columns for exon 3). The (invisible) nodes of the graph represent the set of unique 31-mers (across the 3 genes) starting at the corresponding column of the MSA. Two nodes (representing two consecutive 31-mers in the MSA) are connected by (visible) edges if the corresponding 32-mer, starting at the column index of the first 31-mer, is present in the MSA. Edge flow (line thickness) is proportional to the frequency of the corresponding 32-mer at the underlying column (bounded below). Edge colour indicates the proportions of flow attributable to the 3 genes (for each edge, the absolute count of the corresponding 32-mer at the underlying column can be split into a triplet representing the HLA-A, HLA-B, HLA-C rows of the alignment; the (R, G, B) colour of the edge is obtained by normalizing this triplet). For the purpose of this plot, we treat gap characters as nucleotides. The plots below the graphs show, separately for HLA-A, -B, and–C and separately for each column of the underlying MSA, the (weighted) proportion of 31-mers unique to the locus. For the purpose of these plots, a k-Mer is defined as unique to a locus if it doesn’t occur in the same MSA column of a sequence belonging to another locus. k-Mer weights for each plotted column are proportional to within-locus k-Mer column frequencies. (PNG) [file pcbi.1005151.s001.png]

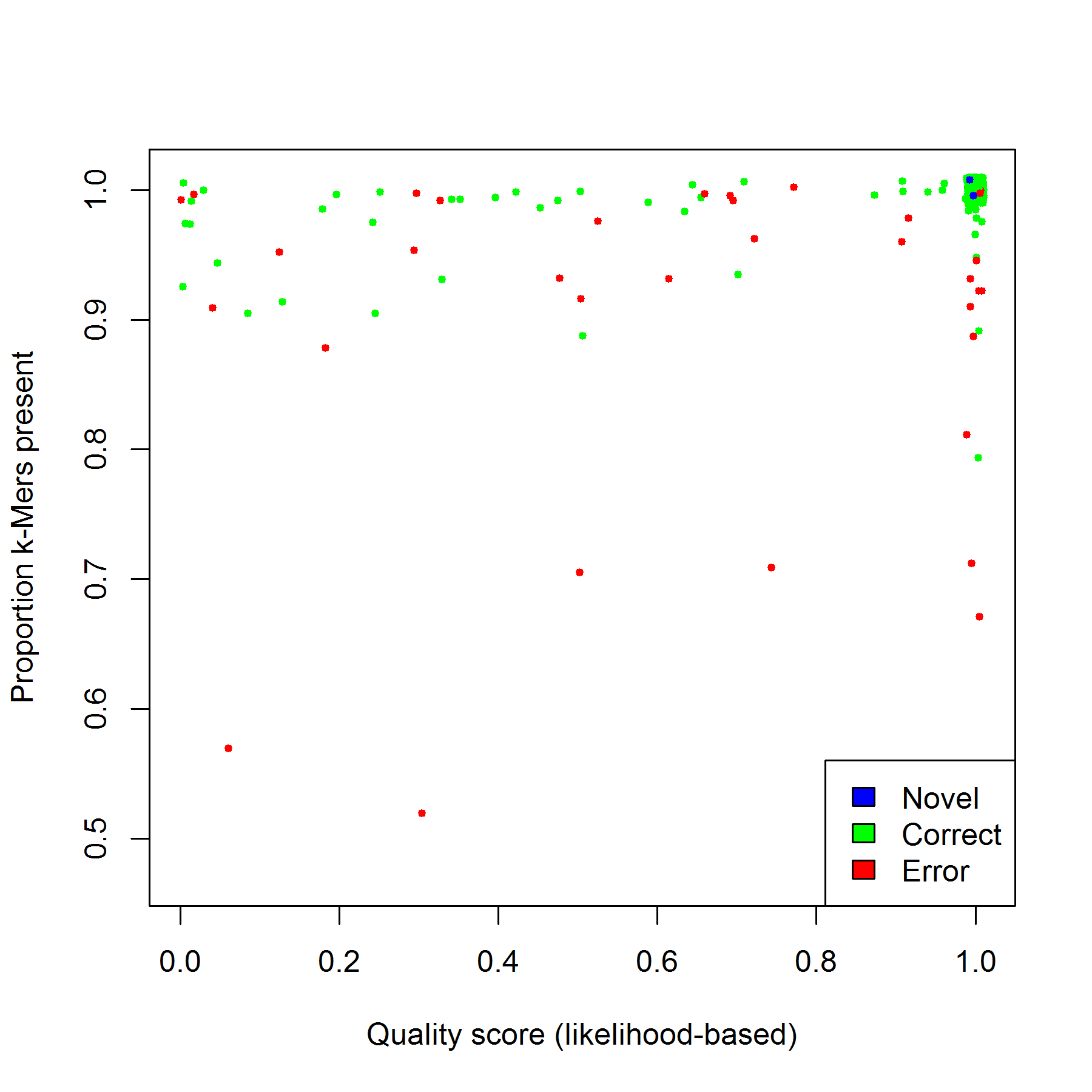

Supplement: S3 Fig — Allele quality score and allele k-Mer coverage at k = 31 for all validated alleles (jittered). The colour of the data points indicates whether an allele was inferred correctly or incorrectly, or whether it was a novel allele (which are counted as ‘incorrect’ for all validation purposes). (TIFF) [file pcbi.1005151.s003.tiff]

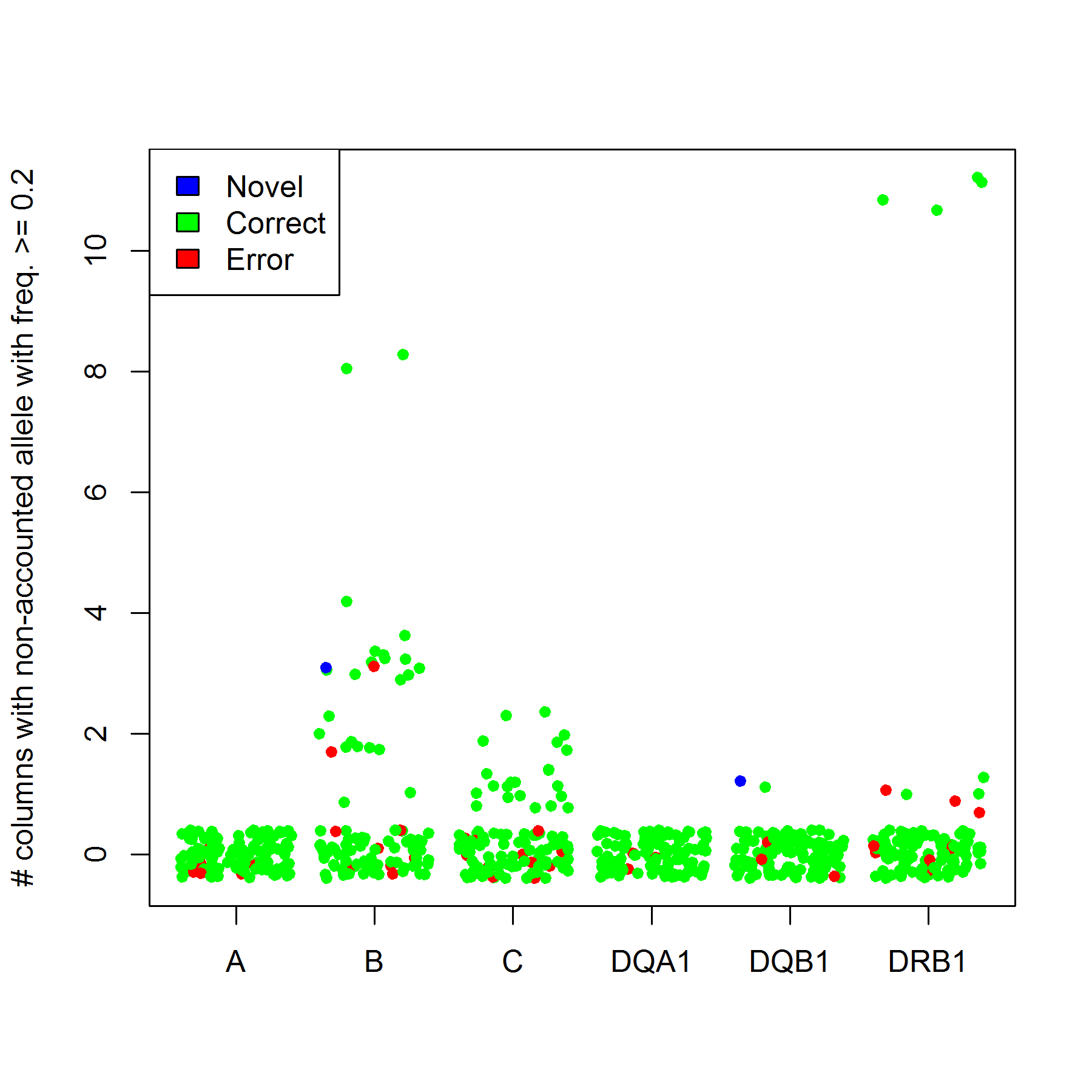

Supplement: S4 Fig — This plot shows, for each inferred allele and stratified by locus, the number of columns in the read-to-graph alignment that contain high-frequency (allele frequency ≥0.2) alleles not accounted for by the inferred (diploid) HLA type. The colour of the data points indicates whether an allele was inferred correctly or incorrectly, or whether it was a novel allele (which are counted as ‘incorrect’ for all validation purposes). (TIFF) [file pcbi.1005151.s004.tiff]
