# Supplementary material for: High-Accuracy HLA Type Inference from Whole-Genome Sequencing Data Using Population Reference Graphs
Source: PLoS Comput Biol. 2016 Oct 28;12(10):e1005151. doi: 10.1371/journal.pcbi.1005151 (PMC5085092; doi:10.1371/journal.pcbi.1005151)
Supplement: S2 Fig — Maximum k-Mer similarity at the peptide binding site (PBS; exons 2/3 for HLA class I, exon 2 for HLA class II) between alleles of different HLA loci, based on k-Mers (k = 25). G group types are defined by PBS sequence. Each cell, in row X and column Y, contains the maximum, over all alleles of locus X, proportion of k-Mers present in any allele of locus Y. This quantity influences the probability of mismapping a PBS read to another locus as exact matching is the first step of the many mapping algorithms, including the one used here. (PDF) [file pcbi.1005151.s002.pdf]

Alleles		A	B	C	DOB	DPA1	DPB1	DQA1	DQB1	DRA	DRB1	DRB3	DRB4	DRB5	E	F	G	H	J	K	L	V
2430	**A**	100%	62%	64%	0%	0%	0%	0%	0%	0%	0%	0%	0%	0%	5%	4%	19%	47%	13%	14%	9%	4%
3084	**B**	76%	100%	100%	0%	0%	0%	0%	0%	0%	0%	0%	0%	0%	6%	4%	19%	35%	15%	8%	9%	4%
2032	**C**	65%	100%	100%	0%	0%	0%	0%	0%	0%	0%	0%	0%	0%	5%	6%	21%	24%	24%	9%	9%	3%
13	**DOB**	0%	0%	0%	100%	0%	0%	0%	0%	0%	0%	0%	0%	0%	0%	0%	0%	0%	0%	0%	0%	0%
37	**DPA1**	0%	0%	0%	0%	100%	0%	0%	0%	0%	0%	0%	0%	0%	0%	0%	0%	0%	0%	0%	0%	0%
193	**DPB1**	0%	0%	0%	0%	0%	100%	0%	21%	0%	38%	24%	5%	21%	0%	0%	0%	0%	0%	0%	0%	0%
51	**DQA1**	0%	0%	0%	0%	0%	0%	100%	0%	0%	0%	0%	0%	0%	0%	0%	0%	0%	0%	0%	0%	0%
459	**DQB1**	0%	0%	0%	0%	0%	12%	0%	100%	0%	31%	17%	2%	11%	0%	0%	0%	0%	0%	0%	0%	0%
7	**DRA**	0%	0%	0%	0%	0%	0%	0%	0%	100%	0%	0%	0%	0%	0%	0%	0%	0%	0%	0%	0%	0%
1374	**DRB1**	0%	0%	0%	0%	0%	34%	0%	23%	0%	100%	100%	39%	76%	0%	0%	0%	0%	0%	0%	0%	0%
59	**DRB3**	0%	0%	0%	0%	0%	18%	0%	17%	0%	100%	100%	15%	50%	0%	0%	0%	0%	0%	0%	0%	0%
16	**DRB4**	0%	0%	0%	0%	0%	9%	0%	10%	0%	100%	61%	100%	50%	0%	0%	0%	0%	0%	0%	0%	0%
21	**DRB5**	0%	0%	0%	0%	0%	18%	0%	10%	0%	100%	61%	22%	100%	0%	0%	0%	0%	0%	0%	0%	0%
13	**E**	13%	10%	13%	0%	0%	0%	0%	0%	0%	0%	0%	0%	0%	100%	0%	3%	6%	0%	0%	0%	1%
22	**F**	8%	15%	13%	0%	0%	0%	0%	0%	0%	0%	0%	0%	0%	0%	100%	2%	3%	1%	1%	1%	0%
50	**G**	33%	29%	29%	0%	0%	0%	0%	0%	0%	0%	0%	0%	0%	3%	2%	100%	6%	7%	7%	4%	0%
12	**H**	84%	50%	38%	0%	0%	0%	0%	0%	0%	0%	0%	0%	0%	6%	3%	5%	100%	9%	6%	4%	2%
9	**J**	23%	28%	39%	0%	0%	0%	0%	0%	0%	0%	0%	0%	0%	0%	1%	9%	9%	100%	4%	10%	0%
6	**K**	24%	14%	16%	0%	0%	0%	0%	0%	0%	0%	0%	0%	0%	0%	1%	7%	6%	4%	100%	3%	0%
5	**L**	16%	19%	21%	0%	0%	0%	0%	0%	0%	0%	0%	0%	0%	0%	1%	4%	4%	10%	3%	100%	0%
3	**V**	8%	11%	7%	0%	0%	0%	0%	0%	0%	0%	0%	0%	0%	2%	0%	0%	2%	0%	0%	1%	100%
