# Supplementary material for: High-Accuracy HLA Type Inference from Whole-Genome Sequencing Data Using Population Reference Graphs
Source: PLoS Comput Biol. 2016 Oct 28;12(10):e1005151. doi: 10.1371/journal.pcbi.1005151 (PMC5085092; doi:10.1371/journal.pcbi.1005151)
Supplement: S5 Fig — The figure shows how insert size and effective fragment length (2 x read length + insert size) differ between cohorts and correctly / incorrectly inferred alleles. (PDF) [file pcbi.1005151.s005.pdf]

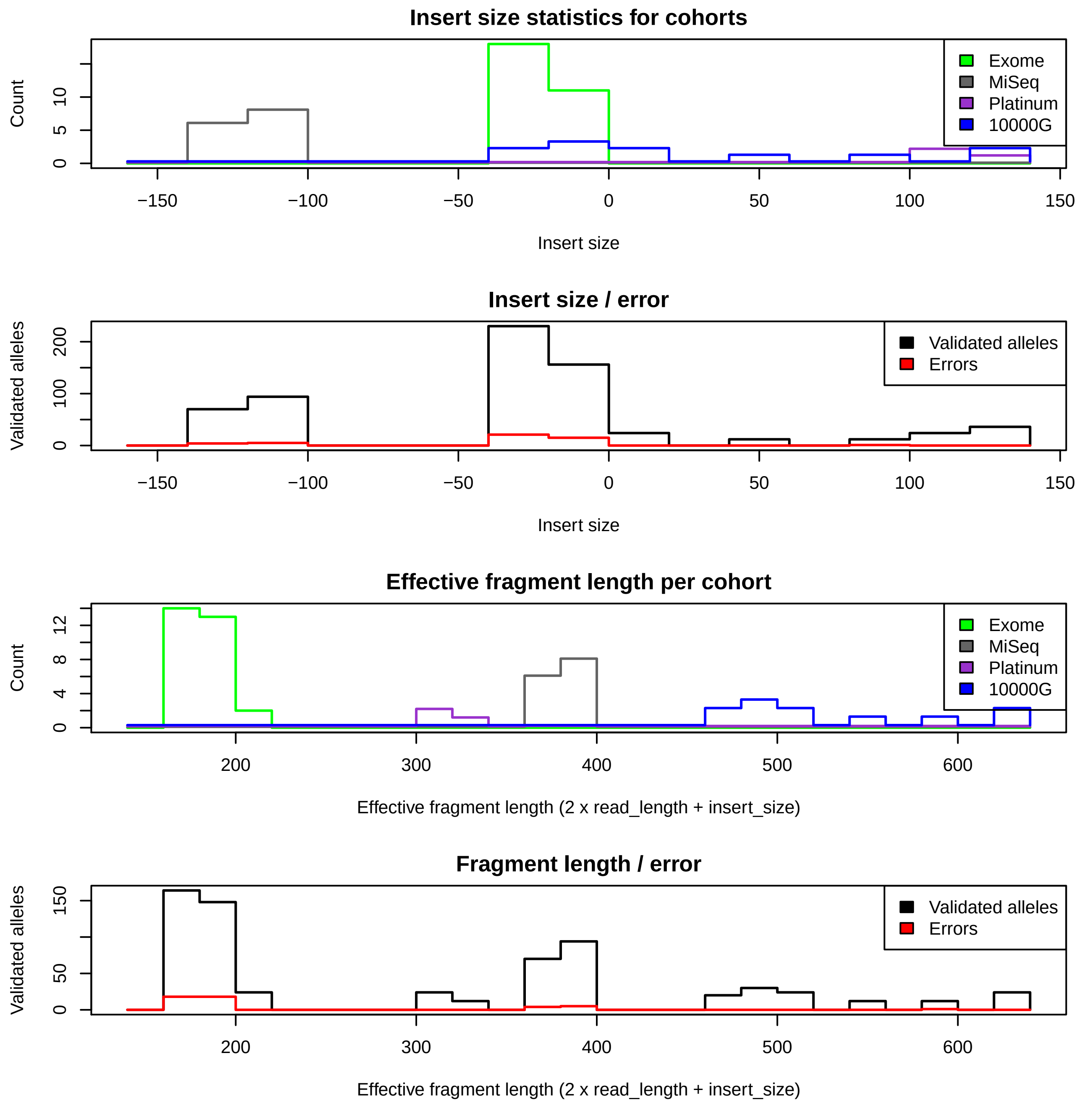

Insert size statistics for cohorts
Count
Insert size
Exome
MiSeq
Platinum
10000G
Insert size / error
Validated alleles
Insert size
Validated alleles
Errors
Effective fragment length per cohort
Count
Effective fragment length (2 x read_length + insert_size)
Exome
MiSeq
Platinum
10000G
Fragment length / error
Validated alleles
Effective fragment length (2 x read_length + insert_size)
Validated alleles
Errors
